# Elective Thoracic Aortic Aneurysm Surgery: A Tertiary Center Experience

**DOI:** 10.7759/cureus.39102

**Published:** 2023-05-16

**Authors:** Abdul Badran, Youssef Elghazouli, Manasi Mahesh Shirke, Mohammed Al-Tawil, Amer Harky, Sunil K Ohri

**Affiliations:** 1 Cardiothoracic Surgery, Southampton General Hospital NHS Foundation Trust, Southampton, GBR; 2 Medicine, Queen’s University Belfast, Belfast, GBR; 3 Surgery, Al-Quds University, Jerusalem, PSE; 4 Cardiothoracic Surgery, Liverpool Heart and Chest Hospital, Liverpool, GBR

**Keywords:** aneurysm, aortopathy, aortic surgery, ascending aortic aneurysm, thoracic aortic aneurysm

## Abstract

Background

A thoracic aortic aneurysm (TAA) is a diseased expansion of the thoracic aorta. There is morbidity associated with a dilated aorta, as well as significant mortality. Open thoracic surgery is the fundamental management for proximal lesions, offering definitive treatment with excellent results. This study aimed to summarize preoperative data and operative outcomes of patients who underwent TAA repair at our institution.

Methods

Data were retrospectively collected from 234 patients that underwent elective open thoracic surgery at University Hospital Southampton for TAA disease, between 2015 and 2019. Demographics, clinical factors, surgical details, as well as outcome measures, were gathered.

Results

There were 166 males and 68 females, with an overall mean age of 66 years. The breakdown of operations comprised 105 aortic roots, 171 ascending aorta, 20 aortic arch, and 12 descending aorta cases. The mean follow-up was 370 days. 30-day mortality was 5.13%. Mortality was associated with female gender, aortic root surgery, and prosthetic valves. Mean aortic diameters at the time of surgery for the non-genetic aortopathy and genetic aortopathy groups were respectively 4.93cm and 4.63cm in the aortic root, 5.56cm and 4.88cm in the ascending aorta, 5.08cm and 3.87cm in the aortic arch, and 6.63cm and 5.50cm in the descending aorta.

Conclusion

Several factors are associated with complications and morbidity, which should be considered when discussing the risks of intervention with patients. There were no neuroprotective strategies that altered post-operative neurological function. Current practice in our unit fits in with current international guidance.

## Introduction

In a healthy adult, the aorta does not exceed 40 millimeters (mm) in diameter. There are a variety of factors that influence the diameter, which include sex, age, body size, and blood pressure [1]. Over the course of life, there is an aortic expansion rate of approximately 0.7mm in women and 0.9mm in men per decade of life [2-5]. Evidence suggests that this slow but gradual dilatation is a natural consequence of aging, associated with greater collagen: elastin ratio, as well as an increase in stiffness and pulse pressure [6].

Aortic aneurysms are considered the second most frequent disease of the aorta, after atherosclerosis, and are described as a pathological dilatation of the aorta [7]. Whilst medial degeneration was originally seen as a non-inflammatory pathology contributing to the aneurysmal formation, more recent findings support the contribution of inflammatory cell infiltration in the disease [8,9]. The thoracic aortic aneurysm (TAA) disease encompasses a lesion in either the aortic root, ascending aorta, aortic arch, or descending thoracic aorta (DTA) with the ascending aorta bring the most common location for TAA [2]. The management of proximal aortic aneurysms - excluding DTA - is carried out by open surgical repair with current guidelines recommending a threshold of ≥ 55mm for surgery indication [2]. Surgery is indicated at lower thresholds in patients with elastopathies or risk factors [10-14]. In the case of a descending TAA, Thoracic Endovascular Aortic Repair (TEVAR) is the first-line treatment and is also indicated for patients with no elastopathy and aneurysms of a maximal diameter of ≥55mm [2]. While the management of TAA patients has been the subject of contention, it is clear that surgery in the ascending thoracic aorta and arch provides the best outcomes. However, operating on these patients after acquired comorbidities carries marked increased surgical risk. The timing and extent of intervention are therefore still variable and debated despite the currently adopted guidelines. We aimed to review the practice in our large tertiary hospital and analyze the outcomes.

## Materials and methods

Data acquisition

**Figure 1:**
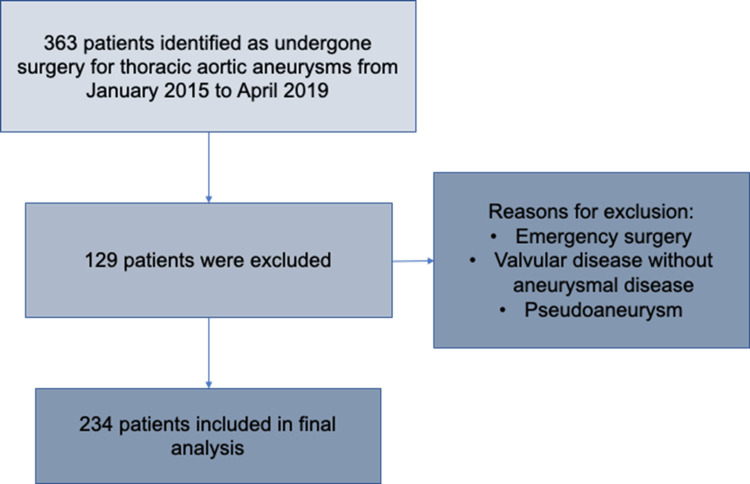
Process of patients' selection

We completed a retrospective cohort study at University Hospital Southampton. We reviewed patient records who underwent surgical repair for TAA from 2015 to 2019. Baseline characteristics including clinical presentation and demographic data and postoperative outcomes were collected and analyzed. Only adult patients (>18 years) who underwent open surgical repair in the elective setting were included. Patients who underwent urgent repair for rupture or dissection were excluded from our analysis. Figure 1 represents the process of inclusion of patients analyzed in this study. Per the mentioned criteria, a final list of 234 adult patients that underwent elective surgery for a TAA at University Hospital Southampton, between January 2015 and April 2019 was analyzed.

Surgical technique

All surgeries were operated by experienced surgeons. Aortic surgery was indicated at an aortic diameter of more than 5.5 cm in isolated aneurysms without risk factors and over 4.5 cm in concomitant cardiac surgery. Patients with connective tissue disease were indicated at thresholds under 5.5cm depending on the disorder, risk factors, and patient choice.

Following median sternotomy, all surgeries were performed under general anesthesia and standard cannulation for the instalment of cardiopulmonary bypass (CPB). In complex cases with prolonged hypothermic circulatory arrest, we used selective antegrade cerebral perfusion. Based on the coexisting indicated surgeries for valvular or coronary disease, further procedures including valve reconstruction/replacement and Coronary Artery Bypass Grafting (CABG) were performed.

Statistical analysis

We used descriptive statistics throughout the study to summarize baseline patient characteristics and procedural outcomes. Data were analyzed using GraphPad Prism (9.0). Categorical variables are presented as frequency distributions (n) and simple percentages (%). To test for difference, the chi-squared test was used for categorical data; T-tests or Mann-Whitney U were used to test for difference between continuous variables depending on the normality of data distribution. Survival probabilities were represented by Kaplan-Meier curves. We categorized patients based on factors related to TAA disease and surgical outcomes and performed a regression analysis to test for predictors of poor outcomes.

## Results

Baseline patient characteristics

**Figure 2:**
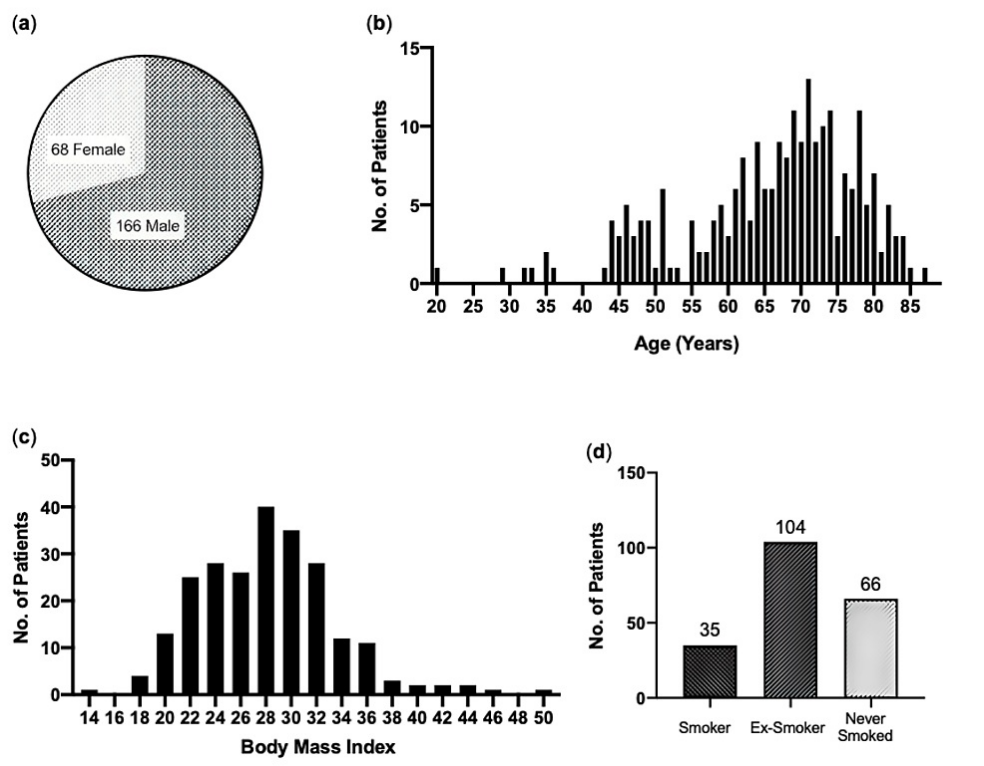
Cohort demographics (a) Gender: Total number of male and female patients making up the cohort. (b) Age: Distribution of patient ages in the cohort. (c) Body Mass Index-BMI: Distribution of patient body mass indexes in the cohort. (d) Smoking: smoking profile of patients in the cohort.

Of the 234 patients included, 68 were female, compared to the 166 males who made up 71% of the cohort (Figure 2a). At the time of surgery, the mean patient age was 66 years, and the median age was 71 years, with 62% of individuals aged 65 years or older (Figure 2b). The mean body mass index (BMI) was 28.0, with 69.5% of the cohort recording a BMI of ≥25, hence clinically overweight (Figure 2c). Of the available records regarding smoking, 67.8% were either current smoker (n=35) or an ex-smoker (n=104), with 66 patients reporting never having smoked (Figure 2d).

**Figure 3:**
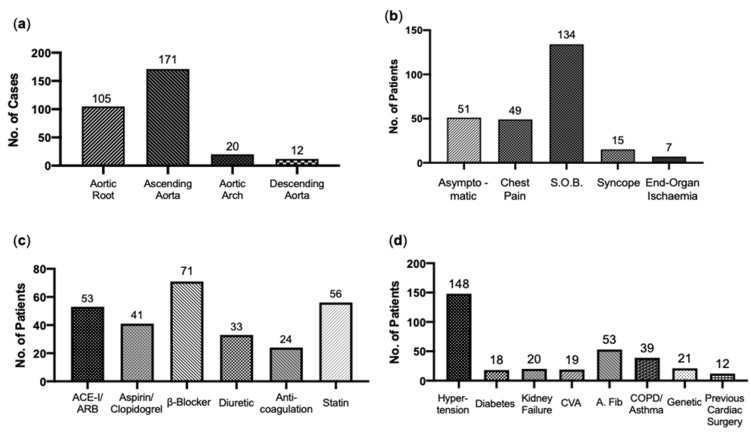
Patient presenting details (a) Aortic segment involvement: Cases presenting with TAA divided by thoracic aortic segment involvement. (b) Presenting symptoms: Reported patient symptoms at presentation. (c) Presenting medications: Medications pre-prescribed at the time of presentation. (d) Past medical history: Previous medical history relevant to aortic disease. S.O.B.: Shortness of Breath, ACE-i/ARBs: Angiotensin-Converting Enzyme Inhibitors/Angiotensin-Receptor Blockers. CVA: Cerebrovascular Accident. A. Fib.: Atrial Fibrillation. COPD: Chronic Obstructive Pulmonary Disease.

With regards to the anatomical location of the aneurysmal lesion, a considerable majority of cases involved the aortic root and/or the ascending aorta (89.6%). One hundred five cases involved the aortic root, 171 the ascending aorta, 20 the aortic arch and 12 the descending aorta (Figure 3a). The majority of patients in the cohort did present with one or more symptoms related to aneurysmal disease, with only 51 patients (21.8%) reported to have been asymptomatic. The most common symptom was shortness of breath, often on minimal exertion, and was noted for 134 patients (57.3%). Forty-nine patients (20.9%) had experienced recent chest pain, with 15 patients (6.4%) experiencing episodes of syncope, and seven (3.0%) patients having had symptoms of end-organ ischemia (Figure 3b). Of the prescription medications taken at presentation, the most common was a β-blocker (30.3%), followed by a statin (23.9%), then an angiotensin-converting enzyme-inhibitor and/or angiotensin receptor blocker (22.6%) (Figure 3c). Of the relevant past medical history, the most common condition was hypertension, noted in 148 patients (63.2%), with atrial fibrillation/flutter second to this, in 53 patients (22.6%). Twelve patients were reported to have had previous open thoracic cardiac surgery (Figure 3d).

Intra-operative details

**Figure 4:**
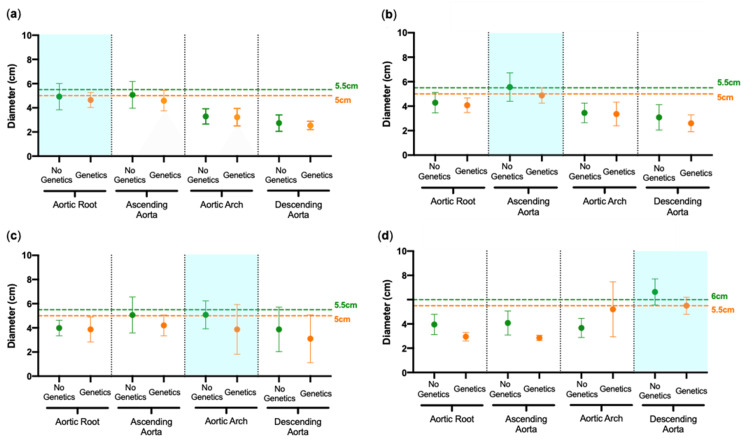
Aortic diameters at the time of surgery Aortic diameter measurements (cm) of cases involving the (a) aortic root, (b) ascending aorta, (c) aortic arch, and (d) descending aorta. Patients are grouped into those with no known genetic aortopathy (green) and those with known genetic aortopathy (orange), each with a corresponding dotted line for the recommended stage of intervention by the European Society of Cardiology and American Heart Association (Mean ± Standard Deviation).

We reviewed aortic diameters pre-operatively and on follow up. Figures 4a-4d depict patients that presented with a TAA involving the aortic root, ascending aorta, aortic arch and descending aorta, showing mean aortic diameters at time of operation. Patients are grouped into those with no known genetic aortopathy (green) and those with known genetic aortopathy (orange), with a corresponding dotted line representing guidelines from the European Society of Cardiology (ESC) and the American Heart Association (AHA) on when to operate. In those that presented with an aortic root aneurysm, the mean root diameter was 4.93cm for the no genetic aortopathy group and 4.64cm for the genetic aortopathy group. In those that presented with ascending aortic aneurysms, these were 5.56cm and 4.90cm, respectively. In aortic arch aneurysm cases, these were 5.08cm and 3.87cm, respectively. In descending aortic aneurysm cases, diameters were 6.63cm and 5.5cm, respectively.

**Figure 5:**
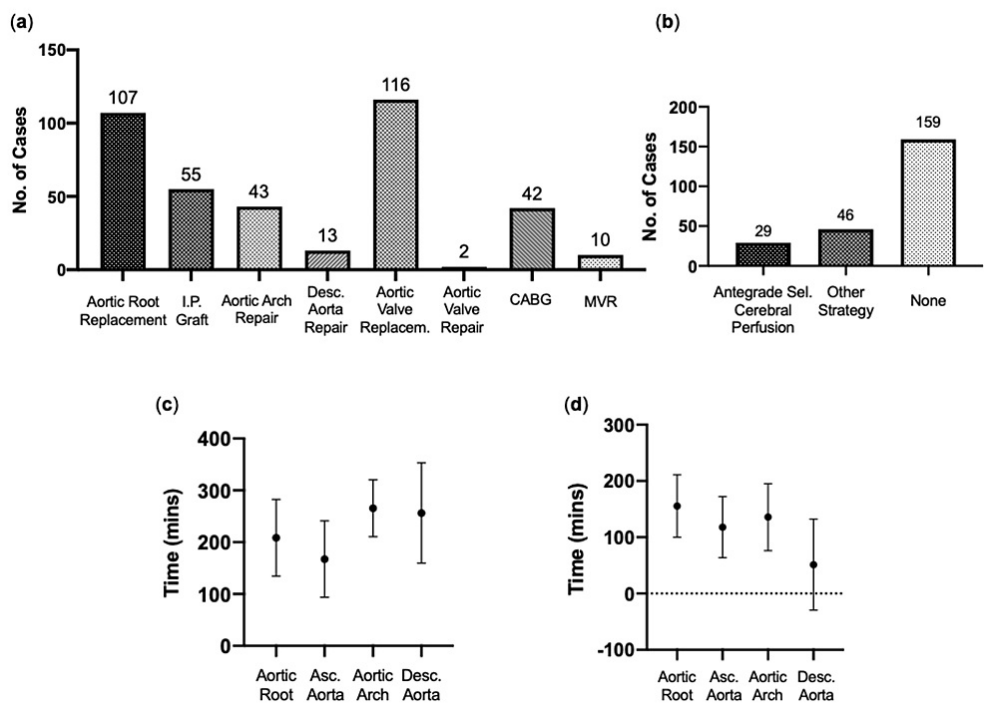
Surgical details (a) Operation Type: Types of operative procedures carried out in cohort. (b) Cerebral Protection: Use of cerebral protection during surgery. (c) Cardiopulmonary Bypass Time: Mean time spent on cardiopulmonary bypass, by location of aneurysmal lesion. (d) Aortic Cross-clamp Time: Mean time spent on aortic cross-clamp, by location of aneurysmal lesion (Mean ± Standard Deviation).

A range of operative types were carried out in these patients, with many individuals requiring additional procedures to those that addressed the aortic lesion (Figure 5a). Of the procedures involving the thoracic aorta, aortic root replacement was carried out in 107 patients (45.7%), making it the most common. This was followed by an interposition graft to the ascending aorta in 55 patients (23.5%), aortic arch repair in 43 patients (18.4%) and repair of descending aorta in 13 patients (5.6%). Aortic valve replacement was carried out in 49.6% of patients (n=116), with aortic valve repair in two patients (0.9%). CABG was required in 42 patients (17.9%), and mitral valve replacement (MVR) was carried out in 10 patients (4.3%) (Figure 4a). One hundred fifty nine procedures (76.9%) utilized no form of cerebral protection, and of those that did, 29 cases (12.4%) incorporated antegrade cerebral perfusion (Figure 5b). Mean time spent on CPB was 208.5mins in cases involving the aortic root, 167.4mins the ascending aorta, 265.5mins the in aortic arch, and 256.3mins the descending aorta (Figure 5c). Mean time spent on aortic cross-clamp was 155.6mins, 118mins, 135.8mins and 51.3mins, respectively (Figure 5d).

Post-operative outcomes

**Figure 6:**
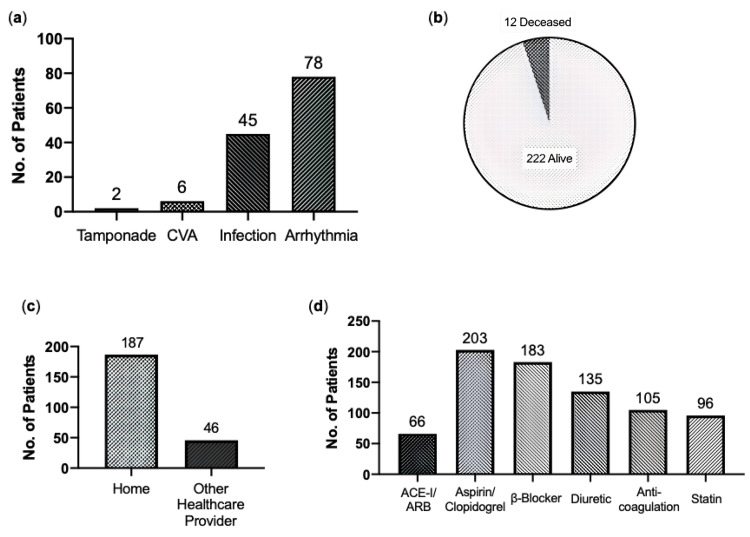
General outcomes and discharge (a) Complications: Number of patients that suffered certain post-operative complications. (b) 60-Day Mortality: Number of patients deceased or alive at 60 days from date of operation. (c) Discharge Location: Number of patients discharged to either home or another healthcare provider for rehabilitation. (d) Discharge Medication: Number of patients prescribed cardiovascular-related medications. CVA: Cerebrovascular Accident. ACE-i/ARBs: Angiotensin-Converting Enzyme inhibitors/Angiotensin-Receptor Blockers

Of the surgical outcomes recorded, 131 patients (56%) suffered a short-term post-surgical complication. Of these, the most common was an arrhythmia (n=78), followed by infection (n=45), cerebrovascular accident (n=6) and tamponade (n=2) (Figure 6a). Overall, 60-day mortality, an indicator of operative-related death, was 5.4% in the cohort, with 12 patients recorded as deceased at 60 days after their operation date (Figure 6b). Discharge location, an indicator of general health, mobility and support need, shows that 80.2% of patients (n=187) were discharged to their usual place of residence, whereas 19.8% (n=46) were referred to a local healthcare provider for ongoing rehabilitation or management (Figure 6c). Of the discharge medication, aspirin/clopidogrel was the most commonly prescribed (n=203, 86.8%), closely followed by a β-blocker (n=183, 78.2%), a diuretic (n=134, 57.3%), an anticoagulant (n=105, 44.9%), a statin (n=96, 41%) and an angiotensin-converting enzyme inhibitors (ACE-i)/angiotensin-receptor blocker (ARB) (n=66, 28.2%).

**Figure 7:**
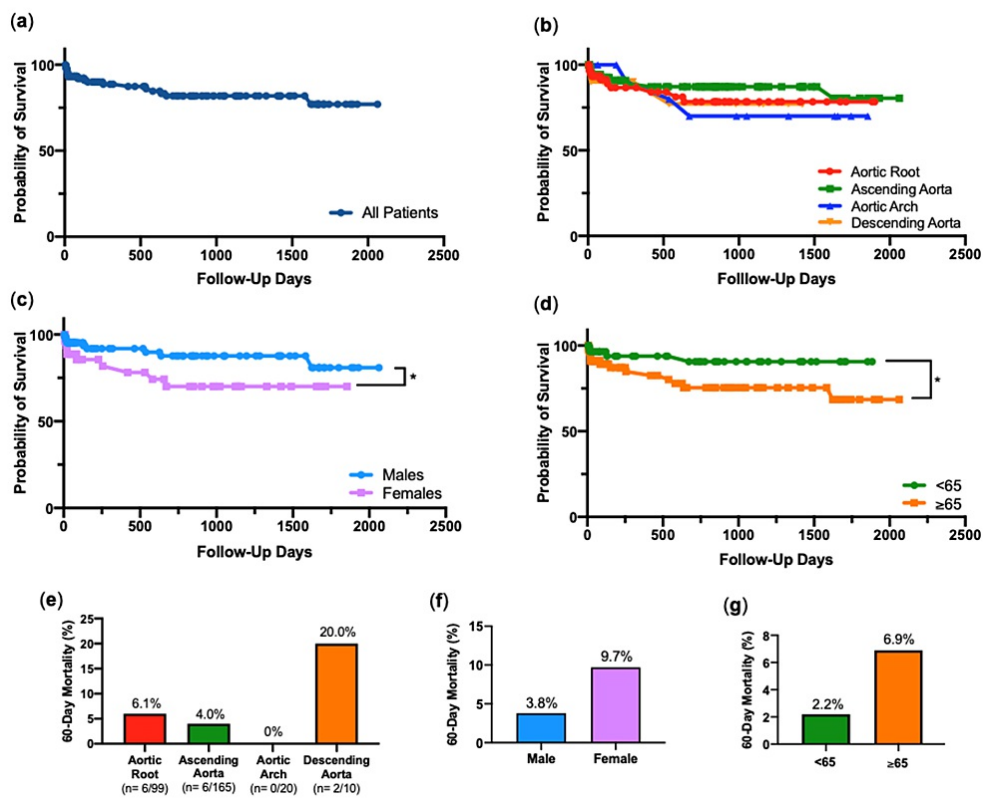
Survival Curves & 60-Day Mortality Kaplan-Meier survival curves for (a) all patients, then grouped into (b) aortic segments involvement, (c) gender and (d) age. *, p<0.05, by log-rank chi-squared test. 60-day mortality grouped by (e) aortic segment involvement, (f) gender, and (g) age.

**Figure 8:**
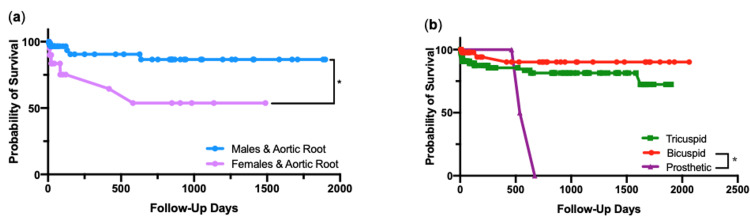
Survival curves Kaplan-Meier survival curves for (a) aortic root involvement grouped by gender, and (b) aortic valve morphology. *, p<0.05, by log-rank chi-squared test.

Figures 7a-7h present Kaplan-Meier survival curves, where the outcome is time until death, using elapsed follow-up days as a measure of time. Figure 7a shows a survival curve for all 234 patients in the cohort. Survival curves grouped into aortic segment involvement showed no statistical significance in survival after log-rank chi-squared test (Figure 7b). Corresponding 60-day mortality for this detailed six deaths from 99 aortic root aneurysm cases (6.1%), six in 165 ascending aorta cases (4.0%), zero in 20 aortic arch cases (0%) and two in 10 descending aorta cases (20%) (Figure 7e). Survival curves categorized by gender show that females in the cohort recorded a significantly lower survival after log-rank chi-squared test (p=0.05) (Figure 7c). 60-day mortality in females was 9.7%, compared to that of in males, 3.8% (Figure 7f). Survival curves grouped by age show that patients aged 65 or over recorded a significantly lower survival compared to those younger, after long-rank chi-squared test (p=0.04) (Figure 7d). 60-day mortality in the ≥65 age group was 6.9%, compared to 2.2% in the <65 age group (Figure 7g). Having carried out further analyses on a number of relationships, a prominent finding was that females that had surgery for aortic root aneurysm involvement fared significantly worse in survival than their male counterparts (p=0.03) (Figure 8a). Categorized by aortic valve morphology, patients with a prosthetic aortic valve fared substantially worse, with statistical significance seen between prosthetic and bicuspid valves, after log-rank chi-squared test (p=0.01) (Figure 8b).

**Figure 9:**
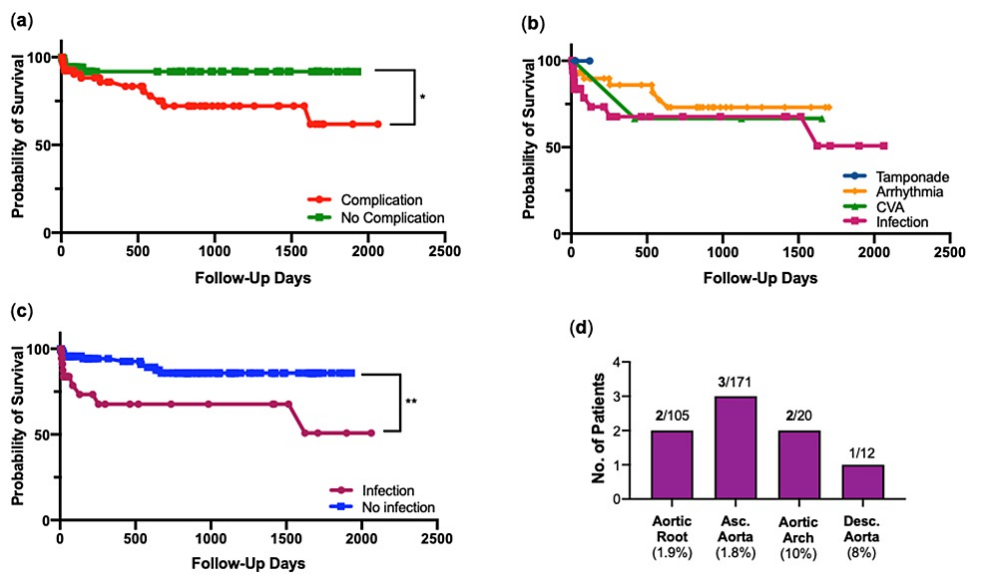
Complications Kaplan-Meier survival curves for (a) post-operative complications vs no complication, (b) each complication and (c) post-operative infection vs no infection. *, p<0.05, **, p<0.005, by log-rank chi-squared test. (d) Post-operative stroke/cerebrovascular accident (CVA) cases by aortic segment involvement.

Further Kaplan-Meier survival curves describing the effects of post-operative complications, show that patients that suffered a post-operative complication fared significantly worse than those that did not (p=0.02) (Figure 9a). When considering survival for individual complications (tamponade, arrhythmia, stroke, infection) in turn there no statistical significance was seen between the groups (Figure 9b). There is an indication in this graph that those that contracted infections post-operatively had a lower survival rate. This is addressed further in Figure 9c, which shows that those who suffered an infection post-operatively had a significantly worse survival than patients that did not contract an infection (p=0.002). A complication of particular interest was post-operative cerebrovascular accident (CVA/stroke), and its associations in the cohort. Regarding the anatomical involvement of the aneurysmal lesion, of the post-operative stroke events, two were out of 105 cases involving the aortic root (1.9%), three were out of 171 cases involving the ascending aorta (1.8%), two were out of 20 cases involving the aortic arch (10%) and one was out of 12 cases involving the descending aorta (8%) (Figure 9d).

## Discussion

The life-threatening risk that TAA disease poses is well-defined, nonetheless, the extensive heterogeneity of the disease poses challenges when making decisions in the management of these patients. While TAA patient management has been contentious in the past, it is widely agreed that surgery offers definitive treatment with excellent results, becoming the mainstay of management. However, operating on these patients after acquiring comorbidities carries distinct surgical risk, thus the timing and the extent of surgical intervention are still variable and debated, despite the availability of internationally adopted guidelines.

Patient demographics: sex differences in TAA disease

It is essential to study the demographic of patients to gain a further understanding of the management of TAA disease. Approximately two-thirds of the patients were over 65 years and 71% were males (Figures 1b, 3a). Studies show that the prevalence of TAA disease does differ greatly between sexes, and throughout all ages, with women reportedly less likely to develop a TAA in their lifetime [15,16]. In our cohort, we found that despite making up less than a third of the patient cohort, female patients had significantly lower survival in this setting (Figure 6c). This is further reflected in 60-day mortality figures, where 9.7% of females died before 60 days, compared to 2.8% in men (Figure 3f). International findings have traditionally shown that mortality rates from cardiovascular disease are greater in men, such as Bots et al. who utilized the World Health Organization (WHO) database from 26 countries [17]. However, our findings are strongly supported in more recent studies assessing gender differences in thoracic aortic aneurysmal disease, which have found TAA outcomes to be worse in women, with women having a threefold greater risk of aortic dissection and rupture [15,18]. Furthermore, acute aortic syndromes have been shown to occur at smaller aneurysm sizes in women, even after the correction of aneurysm size to body surface area [19,20]. In 2019, Boczar et al. presented that aneurysmal growth in TAA patients was more than twice as fast in women, in addition to having a greater aortic stiffness, which is associated with greater TAA expansion [21]. These factors have been demonstrated to contribute to the higher risk of aortic syndromes and deaths seen in women compared to men, and likely to play a role in the outcomes seen in this study.

Patient presentation: aneurysmal location and presenting symptoms

The vast majority of cases had lesions involving the aortic root and/or ascending aorta (Figure 2a). This finding is in line with previous studies [19,22]. The majority of our cohort (78.2%) presented with a symptom possibly attributable to TAA disease (Figure 2b). We found that 62.3% of the reported symptoms were breathlessness, with chest pain second to this at 23.0%. One must consider however that these symptoms could potentially be attributable to other disease processes, but TAA must be considered within the differential in patients with these symptoms.

Timing of elective surgery

Current guidelines recommend intervention at a maximal diameter of 5.5cm for the aortic root and ascending aorta in patients with no known genetic aortopathy, and 5cm in those with a known genetic aortopathy [1,2]. Our results show that for the three proximal aortic segments, our timing of surgical intervention fits in with the current guidelines, with mean aneurysmal size at the intervention recommended cut-off (Figures 3a-3c). For aneurysms of the descending aorta, despite a greater maximal cut-off of 6cm and 5.5cm, respectively, we appear to be operating slightly later (Figure 3d). This is likely due to the nature of descending aneurysms and their common involvement with the abdominal cavity, requiring further consultation and decision-making within a wider disciplinary team. Descending aortic aneurysms had the highest 60-day mortality. Such high mortality can be also explained by the slight delay of surgery in those patients. Still, such results support the endovascular route of intervention as the first line in these patients at indicated thresholds. This also aligns with recent results from Yale, advocating for a left shift to 5 cm as the threshold for surgery indication in patients with ascending TAA [23].

Postprocedural outcomes

Overall, aortic surgery in these patients had acceptable outcomes, considering a 60-day mortality of 5.4%, and when weighed against the threat posed by the condition left untreated, or that of emergency surgery (Figure 5b).

Complications were common in the cohort, with approximately half of the patients experiencing one or more post-operatively (Figure 5a). Unsurprisingly, patients that did experience a complication were associated with a significantly lower chance of survival (Figure 8a). Despite the most frequently occurring complication being arrhythmia (33.3% of patients), cases of arrhythmia were typically resolved with antiarrhythmics pre-discharge and revealed no statistical relationship with survival or 60-day mortality. However, cases of post-operative infection, reported in 19.2% of patients, did present with lower survival when compared to those who did not contract infection (Figure 8c). Infection, predominantly chest infections in this cohort, appear to be the heavy driving force in increased risk seen in these patients and may indicate a need for more extensive follow-up.

We did not find that aortic segment involvement was statistically associated with survival (Figure 6b). When comparing 60-day mortality in these groups, it is important to account for the number of cases involving each segment (Figure 6e). Comparing mortality of the aortic root (n=99) and the ascending aorta (n=165), where the majority of aneurysms were involved, the ascending aorta had a marginally lower mortality rate compared to the root. This is likely due to the greater risk involved with root surgery, nonetheless, outcomes for both appear acceptably safe. While cases involving the aortic arch (n=20) and descending aorta (n=10) were prominently fewer, it is important to note that this challenging procedure had a 0% 60-day mortality. However, two patients (20%) that had descending aortic surgery died at this time. While the numbers are too low to draw statistical conclusions, they may remain an indicator for operative-related survival in these groups.

When analyzing multiple relationships, a key finding was that females who underwent surgery for aortic root aneurysms fared significantly worse in survival in comparison to their male counterparts (Figure 7a). This supports the individual findings in the study that female patients and those undergoing surgery for root aneurysms are higher risk groups, indicating that perhaps these patients should be given greater consideration when deciding on interventional surgery.

Another important relationship was that of aortic valve morphology with survival. Where patients with prosthetic valves were associated with higher risk (Figure 7b). This is likely due to these patients having had a previous open thoracic procedure. They would already have had a lower reserve, so a redo-sternotomy and second cardiac surgery likely added further physiological stress to the body, increasing risk.

Limitations

The major limitation of the current study is its single-center, observational, and retrospective nature. This increases the risk of bias and unmeasured confounders. Additionally, certain parameters such as the 60-day mortality for descending aortic arch surgery have low sample sizes, which may introduce the risk of a Type 2 error. We only analyzed patients who underwent the surgery, patients who offered the surgery and refused were not incorporated in the pre-operative data.

## Conclusions

Overall, operative-associated mortality is relatively low in patients presenting with TAA’s. However, a number of factors are associated with complications and mortality, which should be considered when discussing risks of intervention with patients. With regards to timing of surgery, current practice in our unit does fit in with the current international guidance.
